# Continuous admission to primary school and mental health problems

**DOI:** 10.1186/1471-2458-6-145

**Published:** 2006-06-06

**Authors:** Sijmen A Reijneveld, Carin H Wiefferink, Emily Brugman, Frank C Verhulst, S Pauline Verloove-Vanhorick, Theo GW Paulussen

**Affiliations:** 1 University Medical Center Groningen, University of Groningen, Department of Health Sciences, P.O. Box 196, 9700 AD Groningen, The Netherlands; 2 TNO (Netherlands Organization of Applied Scientific Research) Quality of Life, Division of Child Health, Leiden, The Netherlands; 3 Erasmus University Rotterdam, Academic Hospital Rotterdam-Sophia, Rotterdam, The Netherlands; 4 Leiden University Medical Center, Department of Pediatrics, Leiden, The Netherlands

## Abstract

**Background:**

Younger children in a school class have higher rates of mental health problems if admission to primary school occurs once a year. This study examines whether this relative age effect also occurs if children are admitted to school continuously throughout the year.

**Methods:**

We assessed mental health problems based on parent-reports (using the Child Behavior Checklist, CBCL) and on professional assessments, among two Dutch national samples of in total 12,221 children aged 5–15 years (response rate: 86.9%).

**Results:**

At ages 5–6, we found a higher occurrence of mental health problems in relatively young children, both for mean CBCL scores (p = 0.017) and for problems assessed by child health professionals (p < 0.0001). At ages 7–15, differences by relative age did not reach statistical significance.

**Conclusion:**

Continuous admission to primary school does not prevent mental health problems among young children, but may do so at older ages. Its potential for the prevention of mental problems deserves further study.

## Background

Studies have shown that the youngest children in a school year may be disadvantaged by the educational system [1-5]. Drabman and co-workers showed that in Ohio and Mississippi, USA, the youngest children in a class were referred for academic and behavioral problems more often than their older peers, in a clinical sample aged < 10 years[1]. Goodman and co-workers showed that in the systems in England, Wales, and Scotland, younger children in a school year are at a greater risk of psychiatric disorder than older children, in a large (n = 10,438) community sample aged 5–15 years[2]. Lien and co-workers found that in Oslo, Norway, more peer problems and psychological stress levels, and lower average school grades occurred among the youngest children in a school year, in a sample of 6,752 adolescents aged 15–16 years[3]. Finally, Thompson and co-workers showed that suicide under the age of 20 years in Alberta, Canada, occurred more frequently among those who were younger than their class-mates[4]. An hypothesized explanation for these adverse outcomes among the youngest in a class is that a reduced self-esteem because of relatively lower academic and athletic pursuits is the underlying factor[5].

In the school systems of the countries concerned, all children of a specific age are admitted to school at one moment in the year. As a result of this system, a group of children enters the school at the same moment. In that group the youngest children are almost one year younger than the oldest ones. The youngest can be expected to perform worse in that group at the moment of entry, because they are in an earlier developmental stage, both physically and psychologically. Because of that, Goodman and co-workers proposed to introduce a system that sensitizes teachers to the age position of individual children, thereby reducing the likelihood of unrealistic expectations being placed on younger children[2].

In the Netherlands, children are admitted to primary school throughout the year, on the first day of the month following their 4^th ^birthday. Thus, children enter primary school one by one, which can be expected to sensitize teachers for their relatively young age and to allow them to pay attention specifically to these youngest children. As such, this system realizes the solution proposed by Goodman and co-workers to prevent mental health problems among children, i.e. '.. to sensitise teachers to the age position of individual children within the class, ..' (page 475)[2].

However, in the Dutch system all children enter grade 3 at one specific moment, i.e. after the summer holiday if they are 6 on October 1 of the school year concerned. Children thus stay in grades 1 and 2 for a varying period. Based on this system, children born in October will stay 34 months in grades 1 and 2 (from 1 November till about 1 September – in the Netherlands classes start after the summer holidays someday around September 1), but children born in September only 23 months. All children thus have a similar calendar age at entry, but the latter will be younger at the moment of leaving grade 2, implying that they will never be the oldest in a grade 1/2 class. Retention and acceleration are allowed but no data are available on the proportion of children for whom this goes. This could imply that in grades 1 and 2 fewer problems related to children's relative age occur. Whether the prevalence of mental health problems increases after age 6, remains to be seen. If not, a long-lasting reduction in mental health problems may be reached by admitting children to school continuously instead of once a year.

The aim of this study is to examine whether such a continuous admission to primary school is indeed associated with fewer differences in mental health problems by relative age within a class at ages 5 and 6 (grade 2), and, if yes, whether this effect persists after the age of 6 years.

## Methods

### Participants

We used data on 12,221 children from two sources, both within the framework of the routine preventive health assessments that are provided regularly to all Dutch children. The first source was a cross-sectional national study in 1997 and 1998 on children aged 5–15 (response rate 90.1%; n = 4,480), representative for the Dutch population[6,7]. The second was a randomized controlled trial (RCT) in 2001 and 2002 on the improvement of the early detection of psychosocial problems by child health physicians and nurses (child health professionals, CHP) by a training program, among a national sample (exclusive of the big cities) of children aged 5–6 (response rate 85.2%; n = 7,737)[8]. The latter data source comprised all children that participated in that trial, i.e. the 6,375 children that were analyzed and the 1,477 that were excluded from the primary analysis because of being under treatment or of non-Dutch ethnicity [8]. of 15 of this total group, data on month of birth were missing.

In both studies we obtained participants by means of a two-step procedure. First, Child Health Services were asked to participate. And second, the participating CHS were asked to provide data on a specified number of children. In the cross-sectional sample, this second step concerned a team of child health professionals that were asked to provide data on a sample of 75 children for each age group. In the RCT each participating child health professional was asked to provide data on 50 children for each measurement period, 150 in total. Both studies had been approved by the local Institutional Review Board, including verbal informed consent by parents.

### Measurements

In both studies parents and CHPs filled-out similar questionnaires with the same wording of the items that have been included in this study. Parents first completed the Child Behavior Checklist (CBCL), a well-validated questionnaire on behavioral and emotional problems over the preceding six months[9,10]. It contains 120 problem items on the basis of which a Total Problems score can be computed. Children were allocated to a normal range or a clinical (elevated) range, using the 90th percentiles of the Dutch normative sample for the validated Dutch version[10]. Next, the CHPs took a routine history and physically assessed each child, and then completed the following question: "Does the child have a psychosocial problem at this moment?" (yes or no). If a problem was identified, the CHP was asked to rate its severity as mild, moderate, or severe. All participating CHPs had been trained by the research team on the recording and classification of these problems.

### Analysis

We analyzed whether the occurrence of mental health problems, i.e. mean CBCL Total Problems scores and prevalence of psychosocial problems rated by the CHP as moderate or severe[8], differed by relative age (old = October 1 – January 31, middle = February 1 – May 31, young = June 1 – September 30), by age group (5–6, 7–12 and 13–15 years, i.e. grades 1–2, and grades 3–8 of Dutch primary school; and the first grades of Dutch secondary school, respectively). These analyses were similar to those of Goodman et al. [2], except that these used a psychiatric diagnosis whereas we employed assessment by a CHP. We repeated this with adjustment for differences in background of children in the three relative-age groups, which yielded similar p-values in all cases (not shown).

## Results

Table 1 presents the information on the background of the children in the two samples. We found no differences in background characteristics by relative-age group (range of p-values: 0.062 to 0.850, chi-square tests). Table 2 shows that at ages 5–6 years (grades 1 and 2), differences by relative age in mental health problems existed both for parent-reports on the CBCL (p = 0.017) and for assessments by CHPs (p < 0.0001). At ages 5–6 years, differences by relative age in CBCL Total Problems scores are larger in the RCT data than in the cross-sectional data whereas regarding CHP assessed problems they were slightly larger in the cross-sectional data. For the cross-sectional data, differences at ages 5–6 years were not statistically significant, due to smaller numbers of children involved in that study. At older ages, differences by relative age diminished and statistical significance decreased further.

## Discussion

Our results show that shortly after school entrance the association between the relative age of children in a class and mental health problems was similar in a system in which children were admitted to primary school continuously throughout the year and in a system in which admission occurred once a year[2]. After the age of 6 years, continuous admission seems to have some favorable effects on these relative age effects, though.

### Explanations

The existence of relative age effects in our study may be explained as a lack of support for the contribution of a lower self-esteem of relatively young children to the development of mental health problems among them. The one by one entrance of children in a school class might allow teachers to take into account their young relative age, but this seems to have no favorable effects at that time. An alternative explanation, an earlier physical and psychological developmental stage seems thus to be more likely, because of the independence of this relative age effect from the kind of system of school entry in various countries. We can not exclude some favorable effects of a continuous admission, though, since in our study differences by relative age diminished at older ages whereas they persisted in a system of admission once a year[2]. Finally, it should be realized that children born in October (immediately after the cut-off for admission to grade 3 once a year, October 1) or in one of the following months, stay in grade 1/2 for a longer period than those born in September or the preceding months. The former will thus be the oldest in a grade 1/2 class at some moment, even though all children enter this class at the same age of 4 years. This may affect the self-esteem of the latter group to some extent, and might explain some of the still visible relative age affects.

### Limitations

Our results are unlikely to be biased. Data came from two national studies with both very high response rates [6-8]. Moreover, we used both well-validated questionnaires and professional judgments[9,10], which limits the likelihood of information bias. Finally, we had a very large sample size, slightly larger than that of Goodman et al. [2], and (much) larger than the other studies that showed a relative age effect regarding mental health problems[1,3,4].

However, the two datasets that we studied have been collected for different purposes, one essentially being a prevalence study[6,7], the other being an RCT on the effect of training CHPs to improve diagnostic quality[8]. This is unlikely to have biased our results though, for a number of reasons. Both studies used a very similar methodology, were performed by teams of researchers from the same institute with the same coordinator of the data collection in both studies (SAR), and covered the same population (i.e. the entire Netherlands) though weightings by regions differed somewhat.

Furthermore, the cross-sectional study had a smaller sample size than the RCT which may explain some of the lack of statistical significance for differences by relative age in the former study. However, using only data from the cross-sectional study's absolute differences between the youngest and the oldest in a class also decrease in the higher grades of primary school.

Moreover, country-specific factors may have influenced results, especially the decrease of relative age effects at older ages in our study. It could be that the Dutch educational system in higher grades is effective in preventing problems at those ages. This especially holds for selective acceleration and retention at the entry of grade 3. This process is likely to be facilitated by the opportunity to observe children for two years in a mixed class, followed by a transition of a fixed cohort to the next year, a process in which retention or acceleration is not that much accentuated as it is in 'ordinary' classes.

Finally, we did not obtain a formal psychiatric diagnosis. Therefore, our results need confirmation from studies on the use of the two admission systems within one country, preferably using an experimental design to be able to control for other potential confounders like the type of coaching at school or the degree to which children have attended preschool classes, and with psychiatric assessments.

### Implications

Our study shows the prevalence of mental health problems at ages 5–6 years to be 36% higher for the relatively youngest tertile of children compared to the relatively oldest one. A similar effect exists in other countries, even at older ages. For instance, in the UK the prevalence rate of any psychiatric diagnosis was about 20% higher in the youngest tertile compared to the oldest tertile[2], and in Alberta the number of suicides in the youngest half was about 25% higher than in the oldest half[4]. As it concerns all children in a country, any intervention that decreases the size of this relative age effect may have a relatively large effect. The development of interventions specifically aiming at these relatively young children is thus urgently needed. Moreover, a system of continuous admission deserves further study as our results provide some indications that it has favorable effects at older ages. This may be due to selective retention and acceleration at the transition to grade 3, which is probably facilitated by the existence of mixed grade 1/2 classes with continuous entry. Such a selective transition to grade 3 deserves additional study, especially if it is embedded in a system of continuous admission to grades 1/2.

## Conclusion

Continuous admission to primary school does not prevent mental health problems among young children, but may do so at older ages. Its potential for the prevention of mental problems deserves further study.

## Competing interests

The author(s) declare that they have no competing interests.

## Authors' contributions

SAR had the original idea for the project, wrote the study protocol and coordinated this study and the research part of both studies that provided the data for this secondary analysis. All authors discussed the protocol and formulated the final design. EB and CHW supervised the data collection of the cross-sectional study and the RCT, respectively. SAR did the statistical analyses, which were discussed by all authors. SAR wrote the final manuscript, which was discussed, edited and revised by all authors. All authors read and approved the final manuscript.

## Pre-publication history

The pre-publication history for this paper can be accessed here:



## Figures and Tables

**Table 1:** Background characteristics of children in the two samples

	**Cross-section (n = 4480)**	**RCT (n = 7737)**
Males	2214 (49.4%)	3947 (51.0%)
Ages (grades) *		
- 5–6 years (grade 2)	1435 (32.0%)	7737 (100%)
- 7–12 years (grades 4–8)	1701 (38.0%)	-
- 13–15 years (classes 1–3, i.e. secondary)	1344 (30.0%)	-
Both parents lowly educated **	1514 (33.8%)	1627 (21.3%)
No parent working 17 hours/week or over	331 (7.4%)	305 (3.9%) **
One parent family	372 (8.3%)	499 (6.4%)
No of siblings		
- no	341 (7.6%)	1160 (15.0%)
- 1 sibling	2150 (48.0%)	4084 (52.8%)
- more than 1 sibling	1989 (44.4%)	2493 (32.2%)
Outcomes		
- CBCL	4171 (93.1%)	7737 (100%)
- CHP assessment	4480 (100%)	7737 (100%)

* In the cross-sectional study 6 children (0.1%) were 4 years old and 5 (0.1%) were 16 years old.

** Low = less than 16 years at school (i.e. no high level technical or vocational training for 16+ years). In the RCT, data were missing for 94 children regarding parental education, and for 512 regarding parental employment status.

**Table 2:** Prevalence rates of mental health problems by relative age at school.

**Age-group (years), grade**	**N**	**CBCL Total Problems scores (mean, standard error)**			**P-value**
			
		**Old**	**Middle**	**Young**	
5–6 (grade 2)	9,069	17.10 (0.27)	17.81 (0.27)	18.02 (0.28)	0.017 *
*Cross-section*	*1,332*	*15.64 (0.64)*	*16.03 (0.67)*	*16.27 (0.65)*	0.588 *
*RCT*	*7,737*	*17.35 (0.30)*	*18.10 (0.30)*	*18.32 (0.30)*	0.018 *
7–12 (grades 4–8)	1,602	18.73 (0.67)	18.31 (0.68)	18.77 (0.62)	0.696 *
13–15 (i.e. grades 1–3 of Dutch secondary school)	1,237	16.88 (0.86)	17.11 (0.71)	17.57 (0.80)	0.489 *
					
		Prevalence rates of CHP assessed psychosocial problems ***			
		**Old**	**Middle**	**Young**	
5–6 (grade 2)	9,172	297/2,990 (9.9%)	367/3,064 (12.0%)	422/3,118 (13.5%)	<0.0001 (2x) **
*Cross-section*	*1,435*	*54/485 (11.1%)*	*660/464 (12.9%)*	*74/486 (15.2%)*	0.166/0.059**
*RCT*	*7,737*	*243/2,507 (9.7%)*	*308/2,601 (11.8%)*	*347/2,633 (13.2%)*	<0.0001 (2x) **
7–12 (grades 4–8)	1,701	59/562 (10.5%)	61/535 (11.4%)	67/604 (11.1%)	0.888/0.751**
13–15 (i.e. grades 1–3 of Dutch secondary school)	1,344	52/415 (12.5%)	53/478 (11.1%)	50/451 (11.1%)	0.746/0.513 **

Ages 5–6 concerns the combined cross-sectional and RCT data, the other two ages only concern the cross-sectional data. Data on the CBCL were lacking for 309 children in the cross-sectional study (these did not differ from the remaining regarding any of the variables indicated in Table 1). CBCL = Child Behavior Checklist. CHP = Child Health Professional (doctor or nurse).

* Of Spearman correlation coefficient with relative age.

** Chi-square test and Mantel's test for linearity, respectively, for differences in prevalence rates by relative age.

*** Number of children having a problem/total number of children.
